# 

**Published:** 1854-04

**Authors:** 


### RECEIPTS FOR THE REGISTER.

| | |
|---|---|
| Dr. S: Griffith, Louisville, vol. 7, | $2,00 |
| Dr. C. T. Cushman, Columbus, Ga, vols. 6 and 7,.................... | 4,00 |
| Dr. W. R. Webster, Richmond, Ia. vols. 4 5, 6, and 7,................ | 6.00 |
| Dr. J. W. Keely, Oxford, O, vol. 7 | 2,00 |
| Dr. A. S. Dwyer, Greenfield, O., vol. 7,....................... | 2,00 |
| Dr. J. A. Daugherty, Bowling Green, Ky., vol. 7,..................... | 2,00 |
| Dr. E. Bray, Evansville, Ia., vol 7 | 2,00 |
| Dr. E. Stone, Houston, Texas, vol. 7,.............................. | 2,00 |
| Dr. Henry Olivar, Smithfield, O., vols. 6 and 7,.................... | 4,00 |
| Dr. M. De Camp, Mansfield, O. vol 7,.............................. | 2,00 |
| Dr. H. A. Hartzell, Addison, O., vol. 7,.......................... | 2,00 |
| Dr. S. A. Lynn, Jeffersonville, Ia., vol 7,......................... | 2,00 |
| Dr. C. A. Kells, N. O., La., vol. 7, | 2,00 |
| Dr. E. McCuen, Williamstown, Ky., vol., 7,...................... | 2,00 |
| Dr E. H. Herman, Nashville, Tenn vols. 6 and 7,................... | $4.00 |
| Dr. W. C. Glines, Olive, O., vol. 7, | 2,00 |
| Dr. T. J. Pearce, Decatur, Ia., vol 7 | 2,00 |
| Dr. A. M. Maxwell, Gallion, O vol 7 | 2.00 |
| Dr. W. T. Castner, Clarksville, Tenn., vols. 6 and 7,............. | 4,00 |
| Dr. W. D. Jenks, Frederick City, Md., vols. 5, 6. and 7,........... | 6,00 |
| Dr. Simeon Macy, Metamora, Ia., vol. 7,.......................... | 2,00 |
| Dr. E. B. Edwards, Elkton, Ky., vol. 7,.......................... | 2,00 |
| Dr. J. G. Glenn, Lafayette, Oregon, vol. 7,.......................... | 2,00 |
| Dr. J. R. Garner, Lafayette, Ky., vol. 7,.......................... | 2,00 |
| Dr. B. F. Freeman, Seneca, O. vol. 7,.............................. | 2,00 |
| Dr. A. J. Reeves, Mt. Vernon, O., vol. 7,.......................... | 2,00 |
| Dr. E. C. Sloan, Ironton O., vol. 7, | 2,00 |
| Dr. H. R. Smith, Terre Haute, Ia., vol. 7........................... | 2,00 |
| Dr. J. P. Ulrey, Rising Sun, Ia., vol. 7,.......................... | 2,00 |
| J. D. Chevalier, N. York, advertisement and vol. 7,............. | 12,00 |
| Wrampelmeyer & Otte, adv.,..... | 5.00 |

### A CARD TO THE DENTAL PROFESSION.

The undersigned would respectfully inform the members of the Dental Profession, that they have perfected a plan of mounting teeth on Atmospheric plates, which entirely overcomes all the difficulties heretofore, to wit: the warping of plates and a variety of other difficulties. For which they have obtained Letters Patent, and will furnish office rights on such reasonable terms, that we think no operator will mount a set of teeth without it, after knowing its advantages over the old method.

Those residing in the following states and territories may be supplied with office rights by J. K. Rickey, Keokuk, Iowa, to wit: New York, Pennsylvania, Indiana, Missouri, Illinois, Michigan, Wisconsin, Louisiana, Texas, California, Maine, Vermont, Rhode Island, South Carolina, Iowa, Florida, Minesota, and Oregon Territories. In the remaining Territories, by Crider & Williams, of Lancaster, Ohio.

J. K. RICKCY, Keokuk, Iowa.
H. L CRIDER & D. WILLIAMS, Lancaster. Ohio.

### CHARLES ABBEY AND SONS,

MANUFACTURERS OF

### DENTISTS' FINE GOLD FOIL,

NO. 26, PEAR STREET, PHILADELPHIA.

☞ Orders inclosing CASH solicited, and the Foil will be promptly despactched by Mail, Express, or as otherwise directed.

### CARD TO DENTISTS,

A Dentist in ill health, will sell his fine business, amounting to $3.000 per year; with good will, lease of office, and residence, aituated in a delighiful city, but a few hour communication from New York—by Steambot or Railroad. None need apply unles willing to pay for a well established practice. Address DENTIST,

Care of JAMES TAYLOR, Ed. Dental Register

*CINCINNATI, OHIO.*

### Baltimore College of Dental Surgery.

**Fourteenth Session, 1853-'54.**

CHAPIN A. HARRIS, A. M., M. D.,
Professor of Principles and Practice of Dental Surgery.

THOMAS E. BOND, Jr., A. M., M. D.,
Professor of Special Pathology and Therapeutics.

WASHINGTON R. HANDY, M. D.,
Professor of Anatomy and Physiology.

ALFRED A. BLANDY, M. D.,
Professor of Operative Dentistry.

PHILIP H. AUSTEN, A. M., M. D.,
Professor of Mechanical Dentistry.

REGINALD N. WRIGHT, A. M., M D.,
Lecturer on Chemistry and Metallurgy.

The regular course of Lectures will commence on the 1st of November and close the 1st of March. The Infirmary, Mechanical and dissecting rooms will be open on the 1st Monday in October, during which month there will be occasional lectures from each of the Professors.

Tickets for the Course, $110. Dissecting Ticket, (optional), $10. Diploma Fee, $30. Matriculation Fee, $5.

P. H. AUSTEN, Dean, 84 *Sharp Street, Baltimore.*

---

NINTH ANNUAL ANNOUNCEMENT

OF

### THE OHIO COLLEGE OF DENTAL SURGERY.

**Session of 1853-'54.**

The regular course of lectures in this institution, commences the first Monday of November, and closes on the third Wednesday of February.

**FACULTY.**

JAMES TAYLOR, M. D., D. D. S.
*Professor of the Principles and Practice of Dental Surgery.*

THOMAS WOOD, M. D.,
*Professor of Anatomy and Physiology.*

J. B. SMITH, M. D.,
*Professor of Pathology and Therapeutics.*

JOHN ALLEN, D. D. S.,
*Professor of Operative Dentistry.*

H. R. SMITH, D. D. S.
*Professor of Mechanical Dentistry.*

G. WATT, M. D., *Lecturer on Chemistry.*

Tickets for the course, $100,00 ; Matriculating Ticket, 5,00 ; Diploma Fee, $25 00. Good board can be had at from $2,50 to $3,00 per week. JAMES TAYLOR, *Dean.*

---

### Removal.

W. Z. REES Respectfully informs his friends and patrons, that he has REMOVED his Manufactory to Sixth Street, between Walnut and Vine, South side. A general assortment of DENTAL and SURGICAL INSTRUMENTS always on hand and made to order. Also a general assortment of Pearl Handles and Mirrors always on hand

